# Post‐traumatic stress disorder and major depression among frontline healthcare staff working during the COVID‐19 pandemic

**DOI:** 10.1111/bjc.12340

**Published:** 2021-10-29

**Authors:** Jennifer Wild, Aimee McKinnon, Abbie Wilkins, Haddi Browne

**Affiliations:** ^1^ Department of Experimental Psychology University of Oxford UK

**Keywords:** depression, health care staff, post‐traumatic stress disorder

## Abstract

**Objectives:**

High rates of probable post‐traumatic stress disorder (PTSD) and major depressive disorder (MDD) have been reported for frontline healthcare staff during the COVID‐19 pandemic. However, rates determined by diagnostic assessment are unknown, as are the onset of symptoms and associated index events.

**Methods:**

We assessed frontline healthcare staff with the Structured Clinical Interview for DSM‐5.

**Results:**

Forty‐four percent met criteria for PTSD and 39% met criteria for MDD. Twenty‐four percent reported COVID‐19 trauma as their index event, with the majority of staff reporting trauma that pre‐dated the pandemic. While PTSD was likely to be pre‐existing, MDD was more likely to develop during pandemic working.

**Conclusion:**

These findings indicate the propensity of healthcare staff to experience a range of occupational and personal trauma associated with PTSD and the need to assess index trauma when diagnosing psychopathology in order to best understand the needs of this workforce.

**Practitioner points:**

- We found high diagnostic rates of PTSD (44%) and major depression (39%) among frontline healthcare staff working during the COVID‐19 pandemic.
- Although major depression developed during the pandemic, PTSD was more likely to be pre‐existing.
- When assessing pandemic‐related psychopathology, it is important to assess the onset and index event related to symptoms.
- Healthcare workers appear to have high rates of PTSD related to occupational and personal trauma, which warrants specific focus in service planning.

## Background

The impact of COVID‐19 has been reported to increase risk of mental ill health amongst frontline healthcare staff. Some studies report rates as high as 39% for probable post‐traumatic stress disorder (PTSD) and 43% for probable moderate to severe depression with up to 13% of staff reporting self‐harm or suicide ideation (Greenberg et al., 2021). A recent meta‐analysis estimated symptom rates of PTSD to be 20.2% and 31.1% for depression (Marvaldi, Mallet, Dubertret, Moro, & Guessoum, 2021). Such high rates have led to international prioritization of the mental health of frontline staff delivering care to COVID‐19 patients (Holmes et al., 2020). To date, research into rates of PTSD and other stressor‐related psychiatric disorders amongst healthcare staff during the pandemic has relied on self‐report questionnaires, with no assessment of index events or onset of symptoms. This has made it difficult to determine whether the observed high rates of psychopathology relate to the pandemic, or had an earlier onset. Studies (i.e., Greenberg et al., 2021; Kang et al., 2020; Lai et al., 2020) relate the high rates of probable mental ill health to the COVID‐19 pandemic. In this study, we assess rates of PTSD and major depressive disorder (MDD) amongst healthcare staff with structured clinical interviews. We also assess the index events associated with PTSD symptoms. We hypothesized that rates would be lower than those assessed by self‐report, since such assessments typically over‐estimate rates of psychopathology (Thombs, Kwakkenbos, Levis, & Benedetti, 2018), that the traumatic events associated with PTSD symptoms would be related to COVID‐19 trauma given the literature associating high rates of mental health disorders among healthcare workers with the pandemic, and that the onset of disorders, that is episodes of PTSD and MDD, would be during the pandemic.

## Methods

### Participants

Frontline NHS staff were contacted between July 2020 and March 2021 via four NHS hospital and Ambulance Trusts in England. Participants were recruited via email, flyer, and advertisement during team meetings and were eligible to take part if they cared for or directed care for COVID‐19 patients. One hundred and eighteen people consented to taking part. Fifteen people withdrew after consenting. One hundred and three participants completed screening measures online (55% female, 72% White British, 28% BAME, mean_age_ = 41.28, *SD*_age_ = 8.11). Healthcare workers included 47% ambulance staff (paramedic, advanced paramedic, ambulance support staff), 30% nurses, 10% allied health professionals, 8% clinical hub staff (999, NHS 111), 5% medical doctors, and 1% clinical support staff. Redeployment to a different clinical role was reported by 24%. The range of COVID‐19 patients managed by this sample was 1–1,000 (median = 21–50) and 66% of staff reported that they had directly experienced COVID‐19 deaths (range 1–200 deaths; median = 1–5 deaths).

Participants who scored above clinical cut‐off on either the PTSD Checklist for DSM‐5 (PCL‐5; Weathers et al., 2013) or Patient Health Questionnaire (PHQ‐9; Kroenke, Spitzer, & Williams, 2001) were contacted and completed a diagnostic assessment for both PTSD and MDD over the telephone. Electronic informed consent was obtained from all participants. Ethical approval was granted by the **Removed for review** Research Ethics Committee (R70497/RE001).

### Measures

PTSD symptoms were measured with the PCL‐5. A score of 31 or higher suggests probable PTSD. Depression symptoms were measured by the PHQ‐9, with item nine ‘Thoughts that you would be better off dead, or of hurting yourself’ used to index self‐harm/suicide ideation. A score of 10 or higher suggests a likely diagnosis of MDD. The Structured Clinical Interview for the Diagnostic and Statistical Manual of Mental Disorders, fifth edition (SCID‐5; First, Williams, Karg, & Spitzer, 2016) was administered to assess for PTSD, the index event and MDD. Episodes of MDD that developed during the pandemic were counted as new episodes regardless of whether or not they were recurrent. Trained psychologists administered the SCID‐5 to assess past and current PTSD and MDD. A sample of 10% of assessment recordings were rated by an independent assessor for the presence or absence of PTSD or MDD, demonstrating excellent agreement (Cohen’s κ = 1.0).

### Data analysis

Normality was assessed via inspection of histograms. Skewness and kurtosis values were calculated and all found to be lower than ±1.0. Descriptive statistics are reported regarding rates of PTSD and MDD. To determine whether rates of PTSD and MDD were lower for clinician compared with self‐report‐assessment, we conducted Chi square analyses comparing frequency of PTSD and MDD diagnoses by assessment tool (self‐report vs. clinician‐administered). Chi square analyses were conducted to determine differences in index event characteristics associated with PTSD diagnoses, such as whether PTSD was more frequently associated with COVID‐19‐related or other trauma and whether or not disorder onset was before or during the pandemic.

## Results

The index event characteristics, proportions of individuals meeting diagnostic criteria, and information regarding symptom onset are reported in Table 1. Overall, *N* = 5 (8.9%) participants who scored above clinical cut‐off on the PCL‐5 identified an alternative trauma when the SCID‐5 was administered. These individuals tended to list a series of Criterion A events, then select one they had not selected when they completed the PCL‐5 for the SCID.

**Table 1:** Index event characteristics, rates of PTSD, and MDD positive screens and diagnoses, and details of diagnostic onset

		Percentage	Frequency	Total *N*
*Index event characteristics*				
Index event	COVID related	24	25	103
	COVID unrelated	76	78	103
	Pre‐pandemic^a^	63	65	103
	Post‐pandemic	37	38	103
	Occupational	52	54	103
	Personal	48	49	103
*Rates of positive screens and diagnoses* ^b^				
PTSD	PCL‐5 Positive screen	38	39	103
	SCID Diagnosed	44	45	56
MDD	PHQ‐9 Positive screen	55	57	103
	SCID Diagnosed	39	40	56
Co‐morbid PTSD MDD	PCL‐5 or PHQ‐Positive Screen	54	60	103
	SCID Diagnosed	29	30	103
*Index event characteristics in SCID diagnosed PTSD*				
Index event	COVID related	27	12	45
	COVID unrelated	73	33	45
	Pre‐pandemic	60	27	45
	Post‐pandemic	40	18	45
	Occupational	56	25	45
	Personal	44	20	45
*Onset of symptoms in individuals meeting diagnostic criteria for PTSD or MDD*				
SCID PTSD onset	Pre‐pandemic	47	21	45
	Post‐pandemic	53	24	45
Index event pre‐pandemic: Onset	Pre‐pandemic	85	23	27
	Post‐pandemic	15	4	27
SCID MDD onset	Pre‐pandemic	15	6	40
	Post‐pandemic	85	34	40

Note

PCL‐5 = PTSD Checklist for DSM‐5, PHQ‐9 = Patient Health Questionnaire, SCID = Structured Clinical Interview for DSM‐5.

^a^ Pre‐pandemic was considered to be any date prior to the WHO declaration in March 2020

^b^ Diagnostic interview administered to *N* = 59 above cut off on either screening measure *N = *3 participants with a positive screen were uncontactable for assessment.

The distributions of psychopathology measures in this sample were PCL‐5 (*M* = 27.36, *SD* = 18.67, range = 0–76), PHQ‐9 (*M* = 9.08, *SD* = 5.99, range = 0–25) indicating overall sample means below clinical cut offs. Twenty‐nine percent of the sample endorsed thoughts of self‐harm or suicidal ideation.

Consistent with our hypothesis, self‐report measures tended to overestimate rates of MDD. Of the 56 individuals above cut off the PHQ‐9, 40 met criteria for MDD suggesting that self‐report measures significantly underestimate MDD diagnoses (*χ* ^2^(1) = 7.72, *p* = .005). However, contrary to our hypothesis, of the 39 individuals who were above cut off on the PCL‐5, 44 met criteria for PTSD, suggesting significant underdiagnosis (*χ* ^2^(1) = 5.83, *p* = .016). Some individuals (*N* = 6) who were above cut‐off on the PHQ‐9 and not on the PCL‐5 were found to have PTSD when assessed with the SCID‐5. Contrary to our hypothesis, most index events were unrelated to COVID‐19 and predated the pandemic. There was no greater likelihood of PTSD diagnostic classification for individuals based on whether the index event occurred pre or post pandemic, was occupational or personal, or was COVID related versus unrelated (*p*s > .59). There was no association between self‐harm or suicide ideation and COVID trauma (*p* = .27). For those who met diagnostic criteria, depressive symptoms were significantly more likely to have had their onset during the pandemic than PTSD symptoms (*χ* ^2^(1) = 13.63, *p* < .0001). The majority (85%) of those who reported that their traumatic index event occurred prior to the pandemic reported that their symptoms also began pre‐pandemic, with 15% of the sample experiencing onset during the pandemic.

## Discussion

Our study identified high rates of PTSD and MDD in healthcare staff whose diversity is representative of staff working for the United Kingdom’s National Health Service. Rates of PTSD were consistent with published figures ascertained via self‐report assessment (Greenberg et al., 2021), and rates of MDD were higher than published figures determined by self‐report, as was suicide and self‐harm ideation. Although our sample was characterized by having worked directly with COVID‐19 patients and having experienced a number of COVID‐related deaths, index events were not predominantly COVID related. This indicates the propensity of healthcare staff to experience a range of occupational and personal trauma. Exposure to COVID trauma and working during the pandemic may have precipitated delayed‐onset PTSD for the 15% of individuals who experienced traumatic events prior to COVID, yet developed PTSD during the pandemic.

PTSD diagnostic caseness based on self‐report was lower than rates identified by clinical interview. This is because some individuals who did not self‐report PTSD symptoms over threshold were invited to clinical interview based on their clinically significant depression scores, then met diagnostic criteria for PTSD. This indicates potential missing diagnoses based on self‐report measures for PTSD. The opposite was observed for major depression, with more individuals meeting diagnostic caseness on self‐report measures than by diagnostic interview.

Our study found that the index events associated with PTSD symptoms amongst healthcare workers were roughly evenly split between occupational and personal trauma, suggesting that whilst occupational trauma clearly plays a role in PTSD among healthcare workers, trauma (e.g., physical, sexual assault, childhood sexual abuse) experienced in one’s personal life is also common. Whilst most studies of frontline healthcare workers have failed to document characteristics of trauma associated with PTSD, Maunder, Halpern, Schwartz, and Gurevich (2012) report high rates of childhood sexual abuse, which would be categorized as personal trauma, amongst paramedics. Our study speaks to the importance of assessing and reporting index events associated with PTSD symptoms when investigating symptom rates of PTSD. A small number of participants (*N* = 5) who scored above clinical cut‐off on the PCL‐5 identified an alternative trauma when the SCID‐5 was administered. This small discrepancy underscores the value of clinician‐assessment of criterion A events.

Although our study is the first to assess healthcare staff with structured clinical assessments to determine rates of psychopathology, there are limitations. Our sample was self‐selected, which may have led to a sample of healthcare‐workers with symptoms taking part. However, the overall sample means for PTSD and MDD were below clinical cut‐off, with 42% within the healthy range on both screening measures. This indicates that individuals with low symptom levels also came forward. For practical reasons and to reduce participant burden, we only assessed individuals for PTSD and MDD if they scored above cut off for either screening instrument, rather than assessing the entire sample who consented to the study. As such, our diagnostic results may underestimate the true rates of psychopathology in this group. However, the symptom measures means for those who were not approached for assessment was well within the healthy range (PCL‐5: *M* = 13.02, *SD* = 8.28 and PHQ‐9: *M* = 4.19, *SD* = 2.53). Our results are also consistent with published research of PTSD and MDD assessed by self‐report (Greenberg et al., 2021). Nevertheless, the use of self‐report measures to screen people to determine who should be administered the SCID complicates the ability to compare rates of PTSD and MDD based on self‐report versus clinician interview because the rates based on clinician interview are predicated on an initial self‐report. Whilst clinician‐administered assessments also rely on individuals to self‐report their symptoms, one of the strengths is the scope to clarify the meaning of questions, elicit examples for each criteria, and clarify responses to gain greater certainty of the presence or absence of a symptom. Finally, the distribution of healthcare workers may have influenced the results since research suggests there could be lower rates of mental ill health among medical staff compared to allied health professionals (e.g., Tan et al., 2020). However, Roberts et al. (2021) report rates of 44% for psychological distress for doctors working during the pandemic in emergency medicine, anaesthesia, and intensive care. Future research could compare rates of mental ill health among the different types of healthcare workers who provided direct care to COVID‐19 patients during the pandemic to better ascertain whether some roles are at greater risk of psychopathology and if so, why.

Our study underscores the need to report index trauma events and the onset of symptoms when describing pandemic‐related psychopathology. The high rates of PTSD seen in this and other studies of frontline healthcare workers conducted during the pandemic may, in part, be due to pre‐existing PTSD. Contrary to the pattern observed with PTSD, but in keeping with the pattern of increased depression in the general population during the pandemic (Czeisler et al., 2020; Ettman et al., 2020; Jia et al., 2020), our study found that the onset of major depression was most likely to occur during the pandemic. This is particularly problematic as major depression is unlikely to remit without treatment and is associated with significant and sometimes long‐lasting, decreased levels of functioning and productivity (Novick et al., 2017) as well as risk and safety with consideration of self‐harm and suicide ideation. Future research is needed to investigate the persistence of PTSD and MDD amongst healthcare workers diagnosed during the COVID‐19 pandemic in order to best plan service provision and understand the needs of this workforce.

## Conflicts of interest

The authors declare that they have no known competing financial interests or personal relationships that could have appeared to influence the work reported in this paper.

## Author contribution

**Jennifer Wild:** Conceptualization; Funding acquisition; Methodology; Supervision; Writing – original draft (equal); Writing – review & editing (equal). **Aimee McKinnon:** Data curation (equal); Formal analysis (equal); Investigation (equal); Validation (equal); Visualization (equal); Writing – original draft (equal); Writing – review & editing (equal). **Abbie Wilkins:** Data curation (equal); Investigation (equal); Writing – original draft (equal); Writing – review & editing (equal). **Haddi Browne:** Project administration (equal); Resources (equal); Software (equal); Writing – original draft (equal).

## Data Availability

Data are available upon reasonable request.
